# Regulated Cell Death Pathways in Pathological Cardiac Hypertrophy

**DOI:** 10.31083/j.rcm2510366

**Published:** 2024-10-11

**Authors:** Shengnan Wu, Ding Ding, Deguo Wang

**Affiliations:** ^1^Department of Geriatrics, The First Affiliated Hospital of Wannan Medical College, 241001 Wuhu, Anhui, China

**Keywords:** regulated cell death, cardiac hypertrophy, pyroptosis, autophagy, ferroptosis

## Abstract

Cardiac hypertrophy is characterized by an increased volume of individual cardiomyocytes rather than an increase in their number. Myocardial hypertrophy due to pathological stimuli encountered by the heart, which reduces pressure on the ventricular walls to maintain cardiac function, is known as pathological hypertrophy. This eventually progresses to heart failure. Certain varieties of regulated cell death (RCD) pathways, including apoptosis, pyroptosis, ferroptosis, necroptosis, and autophagy, are crucial in the development of pathological cardiac hypertrophy. This review summarizes the molecular mechanisms and signaling pathways underlying these RCD pathways, focusing on their mechanism of action findings for pathological cardiac hypertrophy. It intends to provide new ideas for developing therapeutic approaches targeted at the cellular level to prevent or reverse pathological cardiac hypertrophy.

## 1. Introduction

Cardiac hypertrophy, an important risk factor underlying cardiovascular diseases, manifests as an increase in cardiomyocyte size rather than number in the adult heart owing to the terminal differentiation of cardiomyocytes shortly after birth [1]. Cardiomyocyte hypertrophy is distinguished by augmented cellular dimensions, heightened protein synthesis, and increased sarcomere tissue. The workload of cardiomyocytes is escalated during cardiomyocyte hypertrophy by thickening the ventricular wall to sustain cardiac pumping, necessitated by augmented peripheral organ load. This is deemed as an adaptive and compensatory response. Cardiac hypertrophy is physiological or pathological, and the underlying molecular mechanisms, prognoses, and other aspects of the two differ greatly. Physiological cardiac hypertrophy is typically reversible [2], marked by increased heart mass and cardiomyocyte size [3]. Conversely, pathological cardiac hypertrophy, which is irreversible, is intertwined with processes, including autophagy and oxidative stress. It is characterized by the elongation of individual cardiomyocytes, cardiomyocyte death, ensuing progression of myocardial interstitial fibrosis, dysfunctional myocardial systole–diastole, perivascular fibrosis, and ultimately, attenuation of the ventricular wall, ventricular chamber dilation, and diminished cardiac output [4, 5]. This leads to several adverse cardiovascular events, such as heart failure, arrhythmias, and death [6]. Therefore, a comprehensive grasp of the molecular mechanisms that underlie pathological cardiac hypertrophy is promising for identifying novel drug targets and enriching therapeutic strategies.

Cell death is a multifaceted and interconnected process. Between 500 and 70 billion cells undergo this process daily within the adult body [7]. Regulated cell death (RCD) under physiological regulation is known as programmed cell death (PCD), which was initially synonymous with apoptosis and is recognized as an active, programmed cellular catabolic process that occurs autonomously without releasing cytoplasmic contents into the extracellular space [8]. However, additional types of RCD have been unveiled recently, including necroptosis and autophagy [9]. Mounting evidence indicates that different RCD and cytokine-type mechanisms regulate pathological cardiac hypertrophy development. Therefore, elucidating the mechanisms of interactions among various cytokines, cell death pathways, and pathological cardiac hypertrophy is expected to facilitate a better understanding of disease pathogenesis and progression.

## 2. Apoptosis

Apoptosis is an evolutionarily conserved and inducible PCD type. In 1972, Kerr *et al*. [10] first proposed the notion of apoptosis and delineated its morphological characteristics, including nuclear and cytoplasmic condensation and degradation of apoptotic bodies by lysosomes. Apoptosis is mainly triggered through extrinsic and intrinsic signaling pathways [11]. Both pathways ultimately converge to caspase-3, which is involved in a protease cascade. Apoptotic caspases can be categorized as initiating caspases (caspases 8 and 9) and executioner caspases (caspases 3 and 7). These proteases are pivotal in cleaving regulatory and structural molecules, ultimately leading to nuclear apoptosis [12].

Extrinsic or cytoplasmic pathways trigger apoptotic signaling by binding to death receptors on the cell surface. Death receptors are transmembrane proteins with cysteine-rich extracellular and intracellular domains containing homologous amino acid residues. The death domain (DD) is crucial in apoptotic signaling due to its protein hydrolysis function [13]. Death ligands, including tumor necrosis factor (TNF-α) and Fas ligand (FasL), bind to death receptors (TNF and Fas), resulting in the formation of the death-inducing signaling complex (DISC) [14, 15]. DISC recruits and activates the initiator caspases, further degrading intracellular components and inducing apoptosis [16].

Intrinsic pathways, or mitochondrial pathways, are triggered by intracellular stressors, including DNA damage, oxidative stress, and loss of survival signals, which cause increased outer mitochondrial membrane permeabilization (MOMP) [17]. This pathway is regulated by the B-cell lymphoma-2 (Bcl-2) family of anti-apoptotic proteins, including the pro-apoptotic effector molecule, Bcl-2-associated X protein (Bax), and the anti-apoptotic protein, Bcl-2 [18]. Upon stimulation, MOMP increases, releasing the pro-apoptotic factor cytochrome c and activating death signaling pathways. The release of cytochrome c from the inner mitochondrial membrane, an important electron transporter in the respiratory chain, blocks electron transfers downstream and compromises the respiratory chain, leading to an accelerated production of the superoxide anion. The Bcl-2 protein can inhibit this process. The binding of cytochrome c to the apoptosis-inducing factor apoptotic protease activating factor-1 (APAF-1) triggers the oligomerization of APAF-1 and the formation of apoptotic bodies that activate caspase-9, which, in turn, initiates the activation of caspase-3, propelling the mitochondrial cascade, and the activated caspases-3 degrades the substrate. The degradation products of the proteins cause changes in mitochondrial permeability, blocking the release of pro-apoptotic proteins from the mitochondria and ultimately leading to apoptosis [19].

Another apoptosis pathway, the perforin/granzyme-mediated signaling pathway, is mediated by cytotoxic T cells to perforate the cells, mainly through granzyme A or granzyme B, leading to apoptosis [20]. Granzyme B, a serine protease, triggers the caspase apoptotic pathway through caspase-3 cleavage and the direct proteolysis of numerous critical caspase substrates [21, 22]. Granzyme A-mediated is caspase-independent, granzyme A enters the target cell through perforin and crosses the mitochondrial membrane, blocking the mitochondrial electron transport chain without increased MOMP [23]; producing reactive oxygen species (ROS) that drive the endoplasmic reticulum (ER) associated SET (Su (var) 3-9, Enhancer of zeste, Trithorax) complex into the nucleus [24]. In the nucleus, granzyme A cleaves the SET complex to activate the nuclease in the complex and produce single-stranded DNA damage that ultimately causes the death of the target cell [25].

Some environmental chemicals, including heavy metals such as arsenic and copper, decompose and enter the natural aquatic environment. When the concentration exceeds the standard, apoptosis is induced in fish cells. Heat shock proteins 70 (HSP70) and metallothionein (MT) can strongly bind to metals but can hardly block apoptosis [26]. Nanoplastic can induce vascular inflammation and apoptosis of endothelial cells* in vitro* [27]. In a follow-up of 34 months in an observational study, patients with carotid plaques developed myocardial infarction and stroke or constituted a composite end point of the high risk for all-cause mortality [28].

## 3. Apoptosis in Pathological Cardiac Hypertrophy

In a healthy heart, apoptosis is extremely low. However, ischemia and resulting hypoxia are well-known promoters of apoptosis. Pressure overload-induced apoptosis is an early pathological feature of cardiac hypertrophy and myocardial remodeling. Once activated, apoptotic cells are replaced by an extracellular matrix, which damages cardiomyocytes, increases interstitial fibrosis, promotes myocardial hypertrophy, and ultimately leads to heart failure [29]—study in mice have confirmed this theory [30]. Accumulating evidence indicates that apoptosis regulates pathological cardiac hypertrophy. Apoptosis promotes hypertrophic cardiomyopathy through cell shrinkage, chromatin compaction, plasma membrane blistering, and nuclear fragmentation [31]. Furthermore, interleukin-18 (IL-18) is a multifunctional pro-inflammatory cytokine and a potent inducer of cardiomyocyte hypertrophy *in vitro*. In a rabbit myocardial model of myocardial infarction, IL-18 enhanced TNF-α-induced cardiomyocyte apoptosis and stimulated caspase-3 activation through an extrinsic pathway, along with enhancing Bax levels in mitochondria and cytochrome c levels in the cytoplasm through an intrinsic pathway, thereby promoting cardiomyocyte apoptosis [32]. Therefore, intrinsic and extrinsic pathways equally regulate apoptosis in cardiomyocytes [33].

Study found that increased expression of Bax, Fas, and FasL promotes persistent cardiomyocyte apoptosis in sinoaortic denervation rats, contributing to cardiac damage [34]. Additionally, a significant elevation in TNF-α levels in hypertrophied cardiomyocytes, with TNF-α inducing the downregulation of cardiomyocyte sarcomere proteins and activation of the extrinsic apoptotic pathway, has been demonstrated *in vitro* [35]. Etanercept, a potent TNF-α inhibitor, competitively binds to TNF-α, thereby blocking its interaction with its receptor [36]. Etanercept treatment significantly reduced post-traumatic cardiomyocyte apoptosis by inhibiting TNF-α-induced cytotoxic reactive oxygen and nitrogen species production [37].

The heart harbors the most mitochondria, comprising approximately 30% of the volume of ventricular cardiomyocytes. Intracellular stressors activate mitochondrial pathways. Apoptosis is correlated with the dysregulated activation of the Wnt signaling pathway [38]. Activation of Wnt/β-catenin signaling triggers the mitochondrial apoptotic pathway. In murine models of transverse aortic constriction (TAC), Wnt/β-catenin mediates the mitochondrial apoptotic pathway by enhancing the activity of the pro-apoptotic protein, Bax, and inhibiting the expression of the anti-apoptotic protein, Bcl-2 [39]. Captopril, an angiotensin-converting enzyme inhibitor, mitigates TAC-induced apoptosis in cardiomyocytes and markedly reduces the expression of cleaved caspase-3 and Bax, while increasing Bcl-2 by inhibiting the Wnt/β-catenin pathway [40]. Elevated Bcl-2 expression mitigates cardiac hypertrophy induced by low ambient temperatures *in vivo* [41]. HSP70 represents a crucial constituent within the heat shock protein family and is key in inhibiting cardiomyocyte apoptosis and myocardial fibrosis [42]. HSP70 is protective in anti-apoptosis as it acts on different links of the apoptosis signaling pathway. Upregulation of HSP70 can promote Bcl-2 expression, reduce Bax expression, and increase the ratio of Bcl-2/Bax, thereby inhibiting cardiomyocyte apoptosis and delaying cardiac hypertrophy [43].

Amphiregulin (AREG), an epidermal growth factor receptor ligand, is widely expressed in cardiomyocytes. Downregulation of AREG in the mouse heart inhibits apoptosis and reduces myocardial hypertrophy by cleaving the pro-apoptotic proteins, Bax and caspase-3 [44]. Study in mice have found that granzyme B is elevated in a mouse model of cardiac fibrosis induced by angiotensin II. Granzyme B deficiency reduces angiotensin II-induced cardiac hypertrophy and fibrosis independent of perforin [45]. Therefore, apoptosis is crucial in pathological myocardial hypertrophy. A specific type of cell apoptosis, pyroptosis, has been recently discovered and confirmed to be intricately involved in the pathophysiology of numerous cardiovascular diseases, and pyroptosis-related regulatory pathways have been implicated in myocardial hypertrophy [46].

## 4. Pyroptosis

The concept of cellular pyroptosis was first introduced by Zychlinsky *et al*. [47] in 1992 by studying macrophages infected with Gram-negative Shigella hosts. They identified a form of cell death distinct from apoptosis, a form of cell death activated by caspase-1. In 2001, Cookson and Brennan [48] termed this inflammatory PCD as pyroptosis. Notably, pyroptosis can be initiated by various pathological stimuli and is characterized by its dependency on caspase-1, which leads to a pro-inflammatory response. Unlike other forms of RCD, pyroptosis involves swift disruption of the plasma membrane integrity, leading to the release of intracellular contents and inflammatory mediators into the extracellular compartment and consequent activation of an intense inflammatory response, which exploits intracellularly generated pores to disrupt electrolyte homeostasis and results in cell death [49]. Ultimately, pyroptosis leads to the release of pro-inflammatory cytokines and is linked to heightened membrane porosity, cell expansion, and DNA damage [50].

The classical pyroptosis pathway is mediated through caspase-1 by gasdermin D (GSDMD). Specifically, pathogen-associated molecular pattern (PAMP) binds to pattern recognition receptors (PRRs). The NOD-like receptor (NLR) family pyrin domain-containing 3 (NLRP3) inflammatory vesicles, a nod-like receptor, recognizes various stimuli, including PAMP [51]. ROS is a key regulator of NLRP3 inflammasome activation [52]. Meanwhile, ROS inhibition reverses NLRP3 inflammasome activation [51]. Following activation, the NLRP3 inflammasome assembles, leading to the activation of caspase-1 [53]. Once activated, caspase-1 cleaves GSDMD at its central junction, initiating partial oligomerization of its N-terminal domain. GSDMD-N release results in pore formation in the cell membrane [54]. In the resting state, GSDMD oligomerization is automatically inhibited by intramolecular binding between the N and C termini [55]. However, cleavage of inflammatory caspase-1 reverses this autoinhibition. Concurrently, activated caspase-1 induces the activation of two major pro-inflammatory cytokines, IL-1β and IL-18, and cleaves the precursors to promote their maturation and secretion. GSDMD is the executor of pyroptosis, and perforation of the plasma membrane by GSDMD-N causes the secretion of mature cellular contents, including IL-1β and IL-18, into the extracellular milieu, which in turn facilitates pyroptosis [56].

## 5. Pyroptosis in Pathological Cardiac Hypertrophy

Many recent studies have demonstrated that pyroptosis is associated with the pathogenesis of pathological cardiac hypertrophy. Silica nanoparticles increased intracellular ROS production and activated the NLRP3/caspase-1/GSDMD signaling pathway in cardiomyocytes, thereby inducing pyroptosis and promoting cardiac hypertrophy [57]. Specifically, NLRP3 inflammasomes facilitate the maturation and release of inflammatory cytokines by activating caspase-1 [58, 59]. Mouse experiments have revealed that large amounts of mtDNA are released into the cytoplasm of oxidatively stressed cardiomyocytes. Oxidative stress activates the cyclic GMP-AMP synthase and stimulator of interferon genes (cGAS–STING) signaling pathway in the hearts of mice with diabetic cardiomyopathy and induces the escape of mtDNA into cytoplasmic lysates. Stimulation of the cGAS–STING pathway subsequently activates the NLRP3/caspase-1/GSDMD-mediated pyroptotic pathway, contributing to advancing diabetic cardiomyopathy [60]. Targeted NLRP3 inhibition and gene silencing blocks caspase-1-dependent pyroptosis in cardiomyocytes and alleviated manifestations of cardiac hypertrophy [61]. Thus, pyroptosis serves a pathological role in cardiac hypertrophy, and inhibition of NLRP3 inflammatory vesicles is a potential target for treating cardiac hypertrophy.

NLRP3/caspase-1/GSDMD is the classical pathway underlying cardiomyocyte pyroptosis. Caspase-1 facilitates cardiomyocyte apoptosis and affects cardiac hypertrophy through its downstream mediator, IL-1β. *In vivo* and *ex vivo* models of cardiac hypertrophy have demonstrated a significant upregulation in caspase-1 and IL-1β expression. Interestingly, combined administration of the caspase-1 inhibitor, Acetyl-Tyr-Val-Ala-Asp-Chloromethylketone (AC-YVAD-CMK), with angiotensin II attenuates the pro-hypertrophic effects of angiotensin II by downregulating caspase-1 and IL-1β levels. AC-YVAD-CMK downregulates cardiac hypertrophy-related markers, including cardiac natriuretic peptide, brain natriuretic peptide, and β-myosin heavy chains, suggesting that caspase-1-regulated pyroptosis is crucial in cardiac hypertrophy. Caspase-1 is involved in cardiac hypertrophy by regulating IL-1β and IL-18 expression [62]. Hence, inhibiting caspase-1 is a potential therapeutic strategy for managing cardiac hypertrophy [63]. GSDMD is a key executor of cellular pyroptosis. DL-3-n-butylphthalide (NBP), a natural compound, reduces GSDMD-N expression in cardiomyocytes by binding to GSDMD-N proteins, a key executor of pyroptosis, and reduces the levels of GSDMD-N in cardiomyocytes by inhibiting the process of GSDMD-N aggregation to form membrane pores; it reduces GSDMD-mediated inflammation to prevent pressure overload-mediated pathological cardiac hypertrophy [64].

## 6. Necroptosis

Degterev *et al*. [65] introduced the concept of necroptosis in 2005. Necroptosis differs from apoptosis in that its progression does not involve caspase activation. Necroptosis is a form of cytolytic cell death resulting in a widespread inflammatory response through the release of endogenous molecules, typically in the form of perforation, rupture of cell membranes, swelling, disintegration of organelles, and leakage of cellular contents, with no obvious morphological changes in the chromatin of the nucleus [66].

Typically, necroptosis is mediated by the interaction of death signaling molecules (TNF-α and Fas) with their corresponding membrane receptors to initiate death receptor signaling [67]. Receptor-interacting serine/threonine protein kinase 1 (RIPK1) and RIPK3 play a key regulatory role. Death receptor signaling activates RIPK3, which contains an N-terminal serine/threonine kinase structural domain and a C-terminal receptor-interacting protein homotypic interaction motif (RHIM) structural domain. RIPK1 interacts with RIPK3, leading to the autophosphorylation of RIPK3. Mixed lineage kinase domain-like protein (MLKL), a functional RIPK3 substrate, binds to RIPK3 through its kinase-like structural domain but lacks kinase activity [68]. Moreover, RIPK3 phosphorylates MLKL, leading to MLKL oligomerization and translocation to plasma membrane endosomes, thereby disrupting cellular integrity [69]. The kinase activity of RIPK3 phosphorylates MLKL and activates RIPK1, RIPK3, and MLKL proteins, forming a necrotic signaling complex, which induces necroptosis. The RIPK1–RIPK3–MLKL pathway is a classical pathway of necroptosis and apoptosis [70, 71].

Caspase-8 triggers apoptosis by inhibiting necroptosis by cleaving RIPK1 and RIPK3 [72]. In death receptor-induced necroptosis signaling, RIPK3, a pivotal mediator of necroptosis, serves as a substrate for caspase-8-mediated proteolysis. Embryonic lethality of caspase-8-deficient mice can be rescued through RIPK3 knockdown, further demonstrating that caspase-8 inactivation promotes necroptosis. Upon inhibition of caspase-8 activity, RIPK1 and RIPK3 interact through the RHIM to form the RIPK1–RIPK3 necroptosis complex [73]. Therefore, two fundamental prerequisites for necroptosis have been identified: (1) the presence of RIPK3 and MLKL within cells and (2) the inactivation of caspase-8 [74].

## 7. Necroptosis in Pathological Cardiac Hypertrophy

Accumulating evidence indicates that necroptosis is important in cardiac hypertrophy, while the inhibition of necroptosis is expected to mitigate the progression of cardiac hypertrophy [75]. The expression of RIPK3 and RIPK1 was elevated in cardiomyocytes after treatment with palmitic acid *in vitro*. Knocking down RIPK3 or RIPK1 attenuated palmitic acid-induced myocardial hypertrophy; meanwhile, Nec-1 (necroptosis inhibitor-1, RIPK1 inhibitor) effectively blocked the expression of hypertrophic marker genes by inhibiting RIPK1. Additionally, caspase-8 activation was reversed, thereby underscoring the significance of necroptosis in mediating cardiomyocyte hypertrophy [76]. Xue *et al*. [77] established a rat model of cardiac hypertrophy by aortic narrowing, revealing a significant elevation in RIPK3 expression in hypertrophied myocardial tissues—the myocardial hypertrophy phenotype was reduced after RIPK3 downregulation. RIPK3 interacts with its downstream target, MLKL, to promote its localization to the cell membrane and increases the influx of intracellular calcium, thus facilitating the progression of myocardial hypertrophy. Similarly, RIPK3 deficiency alleviates myocardial necroptosis, mitigates oxidative stress, and improves myocardial mitochondrial ultrastructure in mice with cardiac hypertrophy. To a certain extent, the downregulation or depletion of RIPK3 attenuates myocardial necroptosis and mitigates myocardial hypertrophy [78, 79].

The RIPK1–RIPK3–MLKL necroptosis complex disrupts cell membrane integrity, causing cell swelling and rupturing, nuclear abscess and lysis, and the release of cellular contents, thereby stimulating innate immune activation and inflammatory responses [80]. Several studies have shown that in diseased myocardial tissues, necroptosis is dependent on the RIPK1/RIPK3/MLKL cascade and is regulated by the RIPK3/(Ca(2+)/calmodulin-dependent protein kinase II (CaMKII) signaling pathway. CaMKII is a newly identified substrate of RIPK3, which mediates necroptosis by activating CaMKII to mediate ischemia and oxidative stress (OS) to trigger myocardial necroptosis [81]. Specifically, RIPK3 can directly phosphorylate CaMKII or indirectly oxidize CaMKII through ROS, which promotes the opening of the mitochondrial permeability transition pore (MPTP), increases mitochondrial membrane permeability, decreases mitochondrial membrane potential, inhibits the mitochondrial oxidative phosphorylation reaction, and promotes ROS production, ultimately causing necroptosis in cardiomyocytes and promoting pathological cardiac hypertrophy [82].

## 8. Ferroptosis

Iron is a key trace element in living organisms, essential for various biological processes, including cellular respiration, DNA synthesis, and redox reactions. However, when iron ions are present in cells above normal levels, they may trigger harmful oxidative stress, damaging cell membranes, proteins, and DNA, ultimately leading to cell death. Initially conceptualized by Dr. Brent R. Stockwell in 2012, ferroptosis represents a distinct form of RCD characterized primarily by lipid peroxidation induced by iron overload [83]. Unlike other RCD pathways, ferroptosis lacks typical features such as cell swelling, plasma membrane disruption, chromatin condensation, and nuclear fragmentation. Instead, it is characterized by diminished or absent mitochondrial cristae, reduced mitochondrial volume, increased density, and outer membrane rupture [84].

Under physiological conditions, iron is primarily absorbed as Fe^2+^, while Fe^3+^ binds to transferrin (TF) in the serum and is recognized by transferrin receptor 1 (TFR1) on the cell membrane. After absorption by TFR1, the STEAP3 metalloreductase in the endosome reduces Fe^3+^ to Fe^2+^. Divalent metal transfer protein 1 (DMT1, or SLC11A2) mediates the entry of Fe^2+^ into the unstable iron pools in the cytoplasm, where it is stored in the iron pool, ferritin, crucial for maintaining iron levels [85]. Glutathione (GSH) and glutathione peroxidase 4 (GPX4) are negative regulators of ferroptosis as they limit ROS production and reduce cellular iron uptake. When GSH is depleted, or GPX4 is inactivated, and the iron pool is overloaded with Fe^2+^, nuclear receptor coactivator 4 (NCOA4) can translocate ferritin into the autophagosome for lysosomal degradation, resulting in the release of free iron [86, 87]. When iron homeostasis is dysregulated, and the balance of iron metabolism is disrupted, massive amounts of free iron are generated through the Fenton reaction to produce large amounts of ROS and lipid peroxide (LPO), which directly damages intracellular substances and destroy structures, leading to rapid cell death, a process known as ferroptosis [88].

## 9. Ferroptosis in Pathological Cardiac Hypertrophy

The involvement of ferroptosis in pathological cardiac hypertrophy remains understudied. However, several studies have identified the modulation of pathological cardiac hypertrophy by ferroptosis. Zhou *et al*. [89] used pyrroloquinoline quinone (PQQ), a natural water-soluble oxidoreductase coenzyme, to treat TAC model mice with cardiac hypertrophy. PQQ suppressed cardiac hypertrophy by inhibiting ferroptosis in hypertrophic cardiomyocytes *in vivo*. Similarly, Xu *et al*. [90] found that nuclear suffix domain 2 (NSD2), a methyltransferase, promotes pathological cardiac hypertrophy by activating ferroptosis signaling in TAC mice. Mixed-spectrum kinase MLK regulates necroptosis in cardiac hypertrophy, and its family member MLK3 is elevated in a pressure load-induced mouse model of cardiac hypertrophy. MLK3 depletion inhibits ferroptosis and expression of oxidative stress-related proteins, thereby exerting a protective role in cardiac hypertrophy [91]. The ferroptosis deterrent protein, cystine/glutamate transporter (xCT), is an Ang II-mediated cardiac hypertrophy inhibitor. xCT inhibition exacerbates cardiomyocyte hypertrophy and elevates Ang II-induced ferroptosis biomarkers, including Ptgs2, malondialdehyde, and ROS [92]. Mouse experiments have proven that ferroptosis exacerbates pathological cardiac hypertrophy [93].

The nicotinamide adenine dinucleotide–sirtuin 1 (NAD–SIRT1) pathway may be an important mechanistic pathway in regulating cardiac hypertrophy through ferroptosis. SIRT1 is a NAD-dependent nuclear histone deacetylase that regulates histone deacetylase activity by deacetylating various histone proteins [94]. The NAD–SIRT1 pathway is closely associated with ferroptosis, with SIRT1 sensitizing cells to ferroptosis by depleting NAD+ and exerting an inhibitory effect on ferroptosis by upregulating GPX4 [95]. α-ketoglutarate increases intracellular NAD levels. Increased NAD activates the expression of SIRT1 and its downstream proteins, including GPX4, and inhibits the ferroptosis of cardiomyocytes, thereby reducing the damage to cardiomyocytes [96].

SIRT1 inhibits ferroptosis by inhibiting oxidative stress. When excess iron is ingested, the body can use ferritin to resist iron toxicity; however, the absence of cardiac ferritin increases ROS production [97]. SIRT1 activation or overexpression *in vitro* can prevent cardiac hypertrophy by blocking pro-inflammatory pathways [98, 99]. Specifically, SIRT1 activation finally alleviates cardiac hypertrophy by inhibiting the ROS-induced TGF1/Sma and mad homolog 3 (Smad3) signaling pathway [100] and inhibiting NLRP3 inflammasome activation [101].

## 10. Autophagy

Autophagy, a cellular self-catabolic process, upholds intracellular stability by degrading organelles, proteins, and other cellular structures through lysosomal degradation, facilitating the removal of damaged organelles and metabolites and providing nutrients to meet cellular requirements. The autophagic process unfolds in two key stages: intracellular targets are enveloped by membrane structures, resulting in the formation of autophagosomes; subsequently, these autophagosomes fuse with lysosomes, leading to the degradation of the enclosed material by autophagic lysosomes. Autophagy is a pivotal regulator of the delicate balance between cell survival and death, exerting the capacity to promote or inhibit cell death [102]. However, excessive autophagy is associated with diverse varieties of caspase-dependent cell death types, termed “autophagic cell death” [103]. Moreover, autophagy is critical for sustaining cellular viability and maintaining energetic homeostasis.

The phosphatidylinositol 3-kinase (PI3K)/protein kinase B (PKB) (Akt)/mammalian target of rapamycin (mTOR) signaling pathway has garnered significant attention recently due to its pivotal involvement in autophagy regulation. PI3K initiates this pathway by facilitating cell membrane formation, subsequently activating its downstream target, Akt, which is translocated to the cell membrane. Akt phosphorylates mTOR to modulate autophagy. mTORC1, an mTOR complex, suppresses autophagy by inhibiting the formation of downstream Unc-51-like kinase 1 (ULK1) and ULK2 autophagy vesicles [104]. Thus, the PI3K/Akt/mTOR pathway exerts a negative regulatory effect on cellular autophagy. The AMP-activated protein kinase (AMPK)/mTOR/ULK pathway is also a crucial regulator of autophagy. AMPK, a key player in energy and metabolic homeostasis, forms a heterotrimeric complex and is widely expressed in metabolic organs [105]. AMPK activation is a positive regulator of autophagy. AMPK promotes autophagy by activating ULK1 directly through phosphorylation or indirectly by inhibiting mTOR, which normally acts as a brake on autophagy [105]. Consequently, cellular autophagy exhibits a dual role, with pathological overactivation and inhibition. Dysregulated autophagy adversely affects cellular metabolism and various organ systems within organisms.

## 11. Autophagy in Pathological Cardiac Hypertrophy

Autophagy removes damaged mitochondria accumulated in hypertrophied myocardium, reducing ROS production in cardiomyocytes. Mitochondrial autophagy in cardiomyocytes is mainly orchestrated through the cytoplasmic E3 ubiquitin ligase, Parkin, and the mitochondrial membrane kinase phosphatase and tensin homolog (PTEN)-induced putative kinase 1 (PINK1) [106]. PINK1 is a serine/threonine kinase localized on the surface of mitochondria and selectively stabilized in the organelle. During mitochondrial depolarization, the electrochemical potential is reduced, and Parkin is recruited to mitochondria for E3 ubiquitin ligase activation [106, 107]. Hence, optimal levels of physiological autophagy are indispensable for maintaining cellular homeostasis. However, excessive or insufficient aberrant autophagic activity can disrupt metabolic equilibrium in cardiomyocytes, facilitating the progression of cardiac hypertrophy.

Numerous studies have demonstrated that autophagy inhibition attenuates pathological cardiac hypertrophy. LncRNA Gm15834 acts as an endogenous sponge RNA for microRNA-30b-3p and is downregulated during cardiac hypertrophy. MiR-30b-3p inhibition enhances autophagic activity in cardiomyocytes, exacerbating cardiac hypertrophy, while miR-30b-3p overexpression inhibits autophagy-induced cardiac hypertrophy by targeting autophagy factors downstream of ULK1 [108]. Xie *et al*. [109] demonstrated that knocking down the immune subunit β5i mitigates cardiac hypertrophy *in vitro*. Cardiomyocytes transfected with siRNA-β5i and treated with chloroquine, an autophagy inhibitor, showed that inhibiting the activity of β5i suppressed cardiac hypertrophy by inducing autophagy. ATG5, a key regulator of autophagy, is a direct target of β5i. Overexpression of β5i promotes the degradation of ubiquitinated ATG5 and inhibits the induction of autophagy, ultimately leading to cardiac hypertrophy. Liu *et al*. [110] used a rat model of cardiac hypertrophy induced by isoproterenol and demonstrated significantly elevated calcium-sensing receptor (CaSR) and autophagy levels in hypertrophied hearts. CaSR inhibition effectively reduces autophagy by inhibiting CaMKK_β_ (eCa2+/calmodulin-dependent protein kinase kinaseβ), thus ameliorating cardiac hypertrophy. Therefore, excessive autophagy contributes to the development of cardiac hypertrophy.

Akt signaling and its downstream targets have been implicated in cardiac hypertrophy. Akt is an important regulator of cardiac hypertrophy, and sustained Akt activation is associated with the PI3K/Akt/mTOR signaling pathway, leading to pathological cardiac hypertrophy associated with mitochondrial dysfunction [111]. Zhang *et al*. [112] showed that calpain 1-mediated mTOR activation mitigated hypoxia-induced cardiac hypertrophy. Zhao *et al*. [113] observed the activation of mTOR and its downstream effectors in aortic arch narrowing-induced cardiac hypertrophy models, along with the downregulation of autophagy markers and reduced myocardial autophagy levels. Protein kinase D, a member of the calmodulin kinase family, regulates autophagy through the Akt/mTOR pathway, participating in cardiac hypertrophy. In contrast, knockdown by the corresponding siRNA inhibited pressure overload-induced cardiac hypertrophy, and myocardial autophagy levels were upregulated. Lower autophagy can lead to cardiac hypertrophy. Thus, myocardial autophagy exerts dual effects on cardiac hypertrophy.

## 12. Interrelation Complexity of Different Cell Death Pathways in Pathological Cardiac Hypertrophy 

Mitochondria are the power source of cells and the main source of ROS generation in cardiomyocytes; thus, mitochondria are crucial in cell death. ROS induces oxidative stress, activates ER stress sensors, transmits apoptotic signals, impairs the antioxidant defense system of cardiomyocytes, and induces cardiac hypertrophy [114, 115]. During necroptosis, ROS production is an effector of the RIP/RIP3/MLKL signaling pathway mediating necroptosis [116]. RIPK3 can indirectly oxidize CaMKII through ROS, opening MPTP, increasing mitochondrial membrane permeability, and causing cardiomyocyte necroptosis [82]. An increase in mitochondrial membrane permeability represents the initial factor of apoptosis. Simultaneously, ROS can induce lipid peroxidation, promoting ferroptosis and autophagy [117, 118]. Autophagy regulation through the ROS signaling pathway can alleviate nicotine-induced cardiac hypertrophy [119].

In ferroptosis, iron can produce excessive ROS through the Fenton reaction. Under physiological conditions, ROS is scavenged by autophagy, a process that can also regulate ferroptosis by removing damaged mitochondria and lipid peroxide [120]. However, excessive autophagy can cause ferritin degradation and induce ferroptosis [121]. Reducing autophagy, ferritin decomposition, and iron content reduction alleviated ferritin-induced oxidative damage and ferroptosis [122]. Additionally, knocking down autophagy-related genes (ATG) increases ferritin content and suppresses ferroptosis. Further, a crosstalk exists between mitophagy and apoptosis. As cell stress increases, mitophagy fails to protect the cell, thus triggering apoptotic signals [123]. Knocking down ATG5 leads to cardiomyocyte apoptosis [124]. The anti-apoptotic protein, Bcl-2, can bind to the autophagy protein, Beclin-1, involved in the formation of autophagosomes and regulates autophagy and apoptosis [125]. When mitophagy is inhibited, damaged mitochondria cannot clear ROS and can directly activate the NLRP3 inflammasome, eventually leading to pyroptosis [126]. Metformin can promote autophagy through the mTOR signaling pathway, inhibit the activation of NLRP3 inflammasome, regulate cell pyroptosis, and alleviate cardiac hypertrophy [127].

## 13. Environmental Triggers of Cell Death in Hypertrophic Myocardium

When the myocardium is stimulated by factors such as hypoxia, mechanical stress, oxidative stress, and neurohumoral overactivation, cardiomyocytes produce and secrete angiotensin II (Ang II), further activating the renin–angiotensin–aldosterone system (RAAS). Continuous Ang II activation can lead to cardiomyocyte apoptosis and induce cardiac hypertrophy [128, 129]. The endoplasmic reticulum (ER) is involved in protein folding, calcium homeostasis, and lipid biosynthesis. During dysregulated protein homeostasis, ER stress-mediated activation of caspase 12 is a marker of maladaptation and an inducer of apoptosis [130]. An increase in soluble free radicals caused by oxidative stress leads to an imbalance in the intracellular redox environment. Myocardial metabolic demand increases, excess free radicals produce damaged cells, and NADPH oxidase produces ROS, which can induce lipid peroxidation through a reaction with polyunsaturated fatty acids in the lipid membrane, ultimately causing ferroptosis of myocardial cells through the Fenton reaction [88]. ROS also stimulates ER stress and promotes apoptosis of cardiomyocytes during hypertension, leading to hypertrophy of cardiomyocytes and activation of myocardial fibroblasts [131, 132].

When the heart is under pressure overload stress, excessive pro-inflammatory factor secretion recruits macrophages into the heart. Cardiac macrophages secrete transforming growth factor (TGF-β), triggering extracellular matrix deposition fibrosis [133, 134]. Changes in stressed ventricular pathological remodeling have been detected in cardiac manifestations of up- or downregulation of Beclin 1 expression, proving that load-triggered cardiac autophagy is a maladaptive response leading to cardiac hypertrophy [135].

Metabolic disturbances can sensitize cardiomyocytes to various forms of cell death, and diabetic cardiomyopathy, a common heart disease in diabetic patients, can lead to cardiac hypertrophy. Notably, when rat cardiomyocytes are stimulated by high glucose, caspase-1, IL-1β, and IL-18 levels increase. The upregulation of microRNAs can induce pyroptosis and promote the occurrence of diabetic cardiomyopathy [136]. Experiments in diabetic mice have confirmed that TNF-α stimulation promotes activation and ubiquitination of RIPK1, followed by the formation of necrosomes, inducing necroptosis of cardiomyocytes, which causes inflammation and cardiac injury [137]. Palmitic acid (PA), a major saturated fatty acid in the blood, induces RIPK-dependent necroptosis, leading to cardiomyocyte hypertrophy upon excessive intracellular lipids accumulation. A crosstalk between ER stress and necroptosis mediates PA-induced cardiomyocyte hypertrophy [76].

## 14. Conclusions and Outlook

**Fig. 1.**
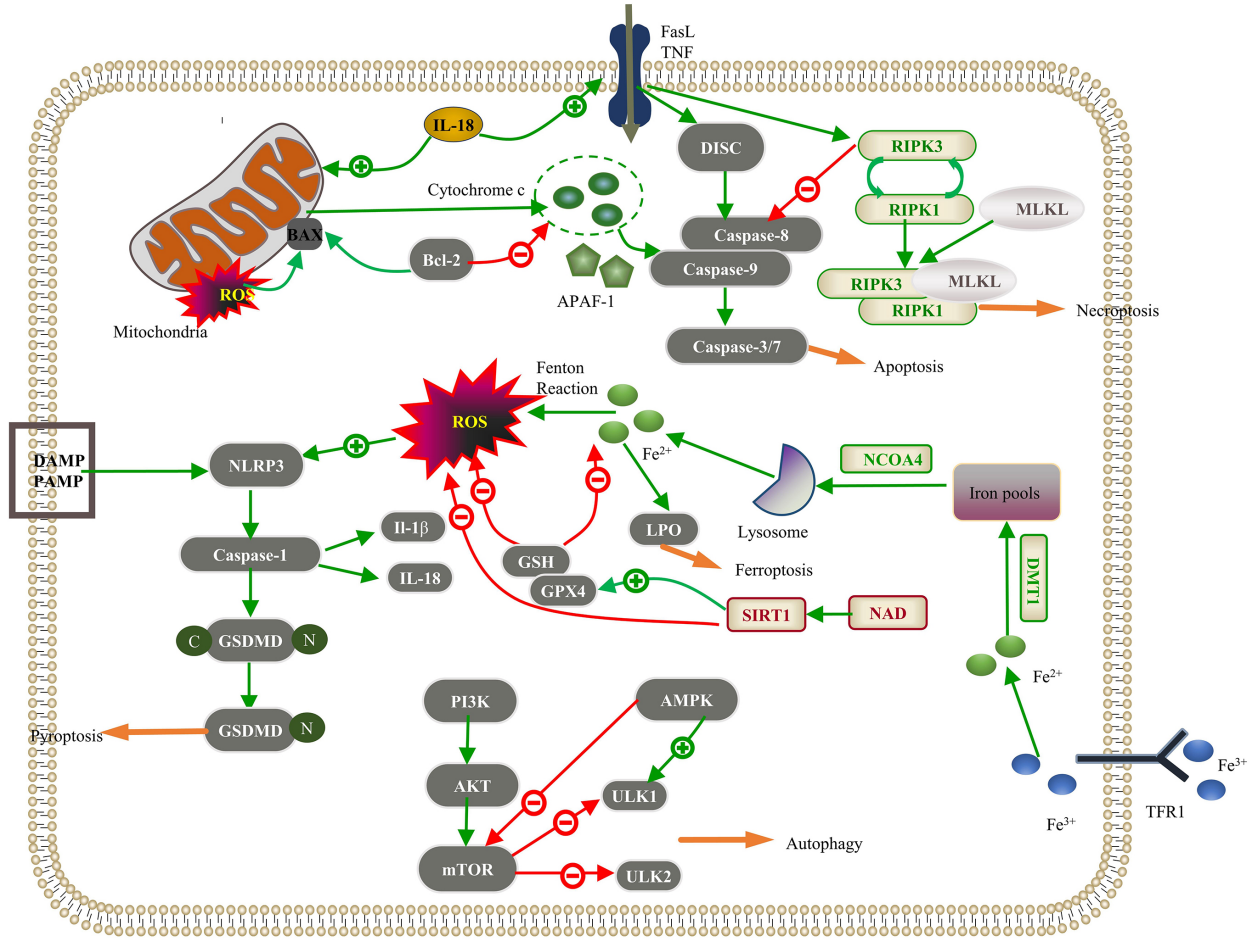
**Regulated cell death (RCD) pathways implicated in pathological cardiac hypertrophy**. Apoptosis: The intracellular damage signal in the intrinsic pathway releases cytochrome c through mitochondrial outer membrane permeabilization (MOMP), which integrates with apoptotic protease activator factor-1 (APAF-1) to activate caspase-9; Bcl-2 inhibits this process. In the extrinsic pathway, the death ligand binds to the death receptor, forming a death-inducing signaling complex (DISC), which activates caspase-8. These jointly activate the “executor” function of caspase-3/7 in apoptosis. Interleukin-18 (IL-18) promotes intrinsic and extrinsic pathways. Necroptosis: The interaction between the death receptor and the membrane receptor initiates the death receptor signal and activates receptor-interacting serine/threonine protein kinase 3 (RIPK3). Upon inhibition of the caspase-8 activity, the interaction between RIPK1 and RIPK3 leads to the autophosphorylation of RIPK3, further activating mixed lineage kinase domain-like protein (MLKL) and leading to MLKL oligomerization. RIPK1, RIPK3, and MLKL proteins form a necrotic signaling complex, which induces necroptosis. Autophagy: The phosphatidylinositol 3-kinase (PI3K)/Akt/mammalian target of rapamycin (mTOR) signaling pathway inhibits downstream Unc-51-like kinase 1 (ULK1) and ULK2 to suppress autophagy, and AMP-activated protein kinase (AMPK) inhibits mTOR to promote autophagy. Pyroptosis: Pathogen-linked molecular patterns (PAMPs) or damage-linked molecular patterns (DAMPs) activate the NLR family pyrin domain-containing 3 (NLRP3), activating caspase-1. Reactive oxygen species (ROS) promote NLRP3 activation, and caspase-1 hydrolyzes gasdermin D (GSDMD) to produce GSDMD-C and GSDMD-N. GSDMD-N polymerizes on the cell membrane to form a nonselective membrane pore; caspase-1 can also cleave IL-1β and IL-18 into mature IL-1β and IL-18, which are released into the extracellular space to exert an inflammatory response. Ferroptosis: Extracellular Fe^3+^ binds to transferrin receptor 1 (TFR1) and is transported to endosomes, where it is reduced to Fe^2+^. Fe^2+^ is transported by divalent metal transporter 1 (DMT1) and stored in the labile iron pool in the cytoplasm. Nuclear receptor coactivator 4 (NCOA4) can transport ferritin to autophagosomes, where lysosomes degrade and release free iron. Excessive iron accumulation in the cell generates massive amounts of lipid peroxides (LPO) through the Fenton reaction. Glutathione peroxidase 4 (GPX4) and glutathione (GSH) can reduce oxidative stress and negatively regulate ferroptosis. SIRT1 inhibits ferroptosis by driving the upregulation of GPX4 and inhibiting ROS. NAD, nicotinamide adenine dinucleotide; BAX, Bcl-2-associated X protein; Bcl-2, B-cell lymphoma 2; SIRT1, sirtuin 1; TNF, tumor necrosis factor.

Emerging evidence underscores the pivotal role of various modes of RCD pathways in orchestrating the intricate network governing the progression of pathological cardiac hypertrophy. Findings from clinical samples, *in vitro* studies, and animal models collectively highlight the involvement of multiple forms of RCD, including apoptosis, autophagy, pyroptosis, ferroptosis, and necroptosis, in regulating pathological cardiac hypertrophy through distinct signaling pathways (Fig. 1). Interconnections between these diverse RCD pathways suggest intricate crosstalk, necessitating comprehensive exploration in the future. Clinical management of cardiac hypertrophy predominantly relies on existing hypertensive medications; however, their efficacy remains limited. A deeper comprehension of the molecular and cellular mechanisms governing RCD is promising for identifying specific drug targets, designing and developing new drugs against these pathways, and integrating drug therapy, gene therapy, stem cell therapy, and other approaches to intervene in cell death pathways. Furthermore, the exact percentage of cardiomyocyte loss attributable to each RCD pathway remains unclear, highlighting a crucial area for future investigation. An in-depth study of the regulatory mechanism of RCD in pathological cardiac hypertrophy offers many opportunities and challenges for future treatment strategies. We anticipate that targeting RCD pathways will emerge as a viable therapeutic strategy for pathological cardiac hypertrophy in the foreseeable future.
